# Online Information of Vaccines: Information Quality, Not Only Privacy, Is an Ethical Responsibility of Search Engines

**DOI:** 10.3389/fmed.2020.00400

**Published:** 2020-08-11

**Authors:** Pietro Ghezzi, Peter G. Bannister, Gonzalo Casino, Alessia Catalani, Michel Goldman, Jessica Morley, Marie Neunez, Andreu Prados-Bo, Pierre R. Smeesters, Mariarosaria Taddeo, Tania Vanzolini, Luciano Floridi

**Affiliations:** ^1^Brighton & Sussex Medical School, Brighton, United Kingdom; ^2^Communication Department, Pompeu Fabra University, Barcelona, Spain; ^3^Iberoamerican Cochrane Center, Barcelona, Spain; ^4^Department of Biomolecular Sciences, University of Urbino Carlo Bo, Urbino, Italy; ^5^Institute for Interdisciplinary Innovation in Healthcare (I3h), Université Libre de Bruxelles, Brussels, Belgium; ^6^Oxford Internet Institute, University of Oxford, Oxford, United Kingdom; ^7^Blanquerna School of Health Sciences, Ramon Llull University, Barcelona, Spain; ^8^Molecular Bacteriology Laboratory, Université Libre de Bruxelles, Brussels, Belgium; ^9^Academic Children Hospital Queen Fabiola, Université libre de Bruxelles, Brussels, Belgium; ^10^The Alan Turing Institute, London, United Kingdom

**Keywords:** search engines, vaccines, health information, information quality, privacy, misinformation, fake news

## Abstract

The fact that Internet companies may record our personal data and track our online behavior for commercial or political purpose has emphasized aspects related to online privacy. This has also led to the development of search engines that promise no tracking and privacy. Search engines also have a major role in spreading low-quality health information such as that of anti-vaccine websites. This study investigates the relationship between search engines' approach to privacy and the scientific quality of the information they return. We analyzed the first 30 webpages returned searching “vaccines autism” in English, Spanish, Italian, and French. The results show that not only “alternative” search engines (Duckduckgo, Ecosia, Qwant, Swisscows, and Mojeek) but also other commercial engines (Bing, Yahoo) often return more anti-vaccine pages (10–53%) than Google.com (0%). Some localized versions of Google, however, returned more anti-vaccine webpages (up to 10%) than Google.com. Health information returned by search engines has an impact on public health and, specifically, in the acceptance of vaccines. The issue of information quality when seeking information for making health-related decisions also impact the ethical aspect represented by the right to an informed consent. Our study suggests that designing a search engine that is privacy savvy and avoids issues with filter bubbles that can result from user-tracking is necessary but insufficient; instead, mechanisms should be developed to test search engines from the perspective of information quality (particularly for health-related webpages) before they can be deemed trustworthy providers of public health information.

## Introduction

The World Health Organization lists vaccine hesitancy as one of the top 10 threats to global health in 2019 (1), requiring ongoing global monitoring. Despite the fact that the 1998 study, which incorrectly suggested that the MMR vaccine could cause autism in children and prompted anti-vaccine beliefs (2), has now been discredited, misinformation and, indeed, disinformation^1^ about vaccines continues to be published on the Internet, perpetuating such beliefs.

It has been suggested that this misinformation plays a role in the current low uptake of vaccines in developed countries (3). Understanding whether this is actually the case, and how to address this issue, is crucial as web-based sources of health information may be instrumental to solve the sustainability challenge currently facing health systems across the globe (4).

The accuracy of information provided by a website is a key indicator of its overall information quality (IQ). The broader aspects of IQ have been the subject of many studies (5), but health IQ and trustworthiness of the sources have only partially been characterized (6). Studies looking at the influence of variations in eHealth literacy levels (7) and trust in different sources of online health information^2^ indicate that the relationship is not linear in all cases, i.e., higher health IQ does not result automatically in higher perceived levels of trustworthiness. For example, in a study by Chen et al. (8), 618 people were recruited to complete a survey which tested their eHealth literacy level and asked them to identify which of 25 sources of health information they used and how much they trusted each source. The study showed that people with lower eHealth literacy were less likely to trust medical websites (typically higher IQ) and more likely to trust social media, blogs, and celebrity webpages (typically lower IQ) (8).

This might seem a spurious result, were it not for the fact that research has shown that those with high eHealth literacy assess more accurately the credibility and relevance of online health information, whereas those with low eHealth literacy often struggle to locate and understand eHealth information (9). This difficulty lowers their self-efficacy (10), distorts their perception of source credibility, and impacts negatively perceived trustworthiness (9), ultimately creating a need for individuals with low eHealth literacy to find an alternative means of determining trustworthiness in online sources of information. One such alternative is social endorsement. Visible social endorsement, e.g., “likes,” (11) enables those with low eHealth literacy to determine trust based on the bandwagon heuristic and assume that, if the source has already been deemed valid by others, then it is safe for them to trust it too (10, 12). Traditional medical websites afford those with low eHealth literacy no such alternative means of determining credibility and trust.

This suggests that those who are more vulnerable to the real-world effects of both disinformation and misinformation (e.g., declining to vaccinate their children) are more likely to rely on online sources with lower health IQ, which are more prone to spread such inflammatory and inaccurate information. Personalization of online search results may favor this phenomenon and lead to a vicious cycle where the more one searches and reads dis- and misinformation about vaccines, the less one finds and reads scientific information on the same topic.

At the same time, there are increasing concerns about the privacy risks associated with Internet search engines storing potentially sensitive and private health information contained within users search histories, combining it with additional information collected for tracking purposes, and using these data for commercial or other purposes (13, 14). This creates a public push back against the idea that search engines or public health providers should interfere in the results people see when searching for health information online. This makes it hard to address concerns about health disinformation/misinformation in a way that is at least socially acceptable if not ideally preferable (15). The UK's National Health Service (NHS) discovered this when it announced it would team up with Amazon to use Amazon Alexa as a voice-activated assistant that would automatically search the NHS website and respond with guaranteed high-IQ content from NHS.UK to user voice queries such as “Alexa, how do I treat a migraine.” This resulted in a public outcry over privacy infringement (16), with advocacy groups raising concerns on handing data from a public healthcare system to a private foreign company. This raises the question whether, in the context of online vaccination information, it is possible at all to balance concerns about user privacy and IQ.

The objective of this study was to address the research question: “What is the current relationship between search engines' approach to privacy and the scientific quality of the information they return?” For this purpose, we used the example of information returned by different search engines after a search on vaccines and autism. The topic was chosen not only because of its primary importance in public health, as described above, but also because the assessment of IQ is straightforward, based on the wide scientific consensus on vaccine safety and of the lack of a causal relationship between vaccines and autism. The study was performed searching the phrase “vaccines autism” in different languages (English, French, Italian, and Spanish) and comparing a wide range of search engines including the main ones (Google, Bing, Yahoo), those branded as “privacy-savvy” (Duckduckgo, Ecosia, Qwant, Mojeek, Swisscows), and some country-specific ones (Arianna and Virgilio, that do not have a specific no-tracking policy).

## Methods

The term “vaccines autism” was used (in French “vaccins autism,” in Italian “vaccini autism,” in Spanish “vacunas autism.” Searches in English were done from Falmer, Sussex, United Kingdom; in Italian from Urbino, Italy; in French from Bruxelles, Belgium; and in Spanish from Barcelona, Spain. Each search was done using a logged-out Chrome browser cleared of cookies and previous search history so that the only identification was the IP address and its geolocalization. Additionally, when available, the local version of each search engine was used (e.g., Google.co.uk and Google.it). For searching Google.com, automatic redirection to Google.co.uk was avoided by using the URL Google.com/ncr (no-country-redirect).

The first 30 URL results from each search engine result page (SERP), excluding those marked as advertisements, were transferred to a spreadsheet. Pages that contained no information, aggregators, and indexes were excluded. Websites were then visited and the content of each page was coded as vaccine-positive, -negative or -neutral, depending on the stance taken on the connection between vaccines and autism.

Webpages recommending vaccination and/or negating the link with autism were coded as “vaccine-positive.” Those promoting vaccine hesitancy, cautioning about the risk of autism or openly anti-vaccine, were coded as “vaccine-negative.” Additionally, webpages that claimed further studies needed to be conducted to clarify the link between vaccines and autism were also coded as “vaccine-negative,” as previous research (17) has shown that users perceive this as confirmation of the fact that vaccine safety has not been proven. Webpages simply reporting the history of the anti-vaccine movement or a related legal debate were coded as “vaccine-neutral.” Examples of positive, negative, and neutral webpages are provided in Table 1.

**Table 1:** Example of classification of webpages.

**Positive stance on vaccines**
https://www.historyofvaccines.org/content/articles/do-vaccines-cause-autism https://www.nhs.uk/news/medication/no-link-between-mmr-and-autism-major-study-finds/ https://www.nhs.uk/conditions/vaccinations/mmr-vaccine/ https://www.autism.org.uk/get-involved/media-center/position-statements/mmr-vaccine.aspx https://kidshealth.org/en/parents/autism-studies.html https://vaccine-safety-training.org/mmr-vaccine-increases.html https://www.nejm.org/doi/full/10.1056/NEJMoa021134 https://www.ncbi.nlm.nih.gov/pubmed/30986133 https://www.ncbi.nlm.nih.gov/pubmed/15366972
**Negative stance on vaccines**
https://www.thelancet.com/journals/lancet/article/PIIS0140673605756968/fulltext www.whale.to/vaccine/vaccine_autism_proven.html www.vaccineriskawareness.com/Infant-Vaccines-Produce-Autism-Symptoms-In-Primates https://leftbrainrightbrain.co.uk/2014/07/17/more-of-that-vaccineautism-research-that-doesnt-exist/ edition.cnn.com/2011/HEALTH/01/05/autism.vaccines/index.html https://www.coasttocoastam.com/show/2018/02/21 nocompulsoryvaccination.com/2014/08/22/vaccine-autism-cover-up/ https://www.newswars.com/vaccine-autism-questioned-by-doctor-congressman-elect/ vaxtruth.org/2011/08/vaccines-do-not-cause-autism/
**Neutral stance on vaccines**
https://www.physicsforums.com/threads/vaccine-and-autism-link.880852/ www.discovermagazine.com/2009/jun/06-why-does-vaccine-autism-controversy-live-on https://www.ecso.org/news/autism-charity-founder-anti-vaccination-campaigner/ https://www.theguardian.com/society/2019/jun/01/professor-who-links-vaccines-to-autism-funded-through-university-portal https://www.independent.co.uk/news/world/americas/trump-vaccines-autism-links-anti-vaxxer-us-president-false-vaccine-a8331836.html https://www.ncbi.nlm.nih.gov/pmc/articles/PMC2376879/

Coding was completed by two raters for each language, and inter-rater agreement was calculated with GraphPad, which uses equations 18.16–18.20 from Fleiss (18). On a sample of 59 webpages in English, agreement was 85%, with a Kappa of 0.669 (standard error, 0.077) and a 95% confidence interval from 0.518 to 0.820, a strength of agreement considered to be “good” (18). In Italian, agreement was 90%, with a Kappa of 0.818 (standard error, 0.067) and a 95% confidence interval from 0.687 to 0.950, a strength of agreement considered to be “very good.” In Spanish, agreement was 83%, with a Kappa of 0.609 (standard error, 0.098) and a 95% confidence interval from 0.418 to 0.801, a strength of agreement considered to be “very good.” In French, agreement was 89% with a Kappa of 0.746 (standard error, 0.091) and a 95% confidence interval from 0.568 to 0.924. Disagreements were resolved by further discussion with a third rater to reach an agreement.

When frequencies of vaccine-negative webpages were compared across different search engines, we used a two-tailed Fisher's test corrected for multiple comparison using the method of Benjamini, Krieger, and Yekutieli and a false discovery rate of 5%. Statistical analysis was performed using GraphPad Prism 8.3.0 for Windows (GraphPad Software, San Diego, CA).

The list of URLs for all searches and their coding is provided in Supplementary File 1.

## Results

**Figure 1:**
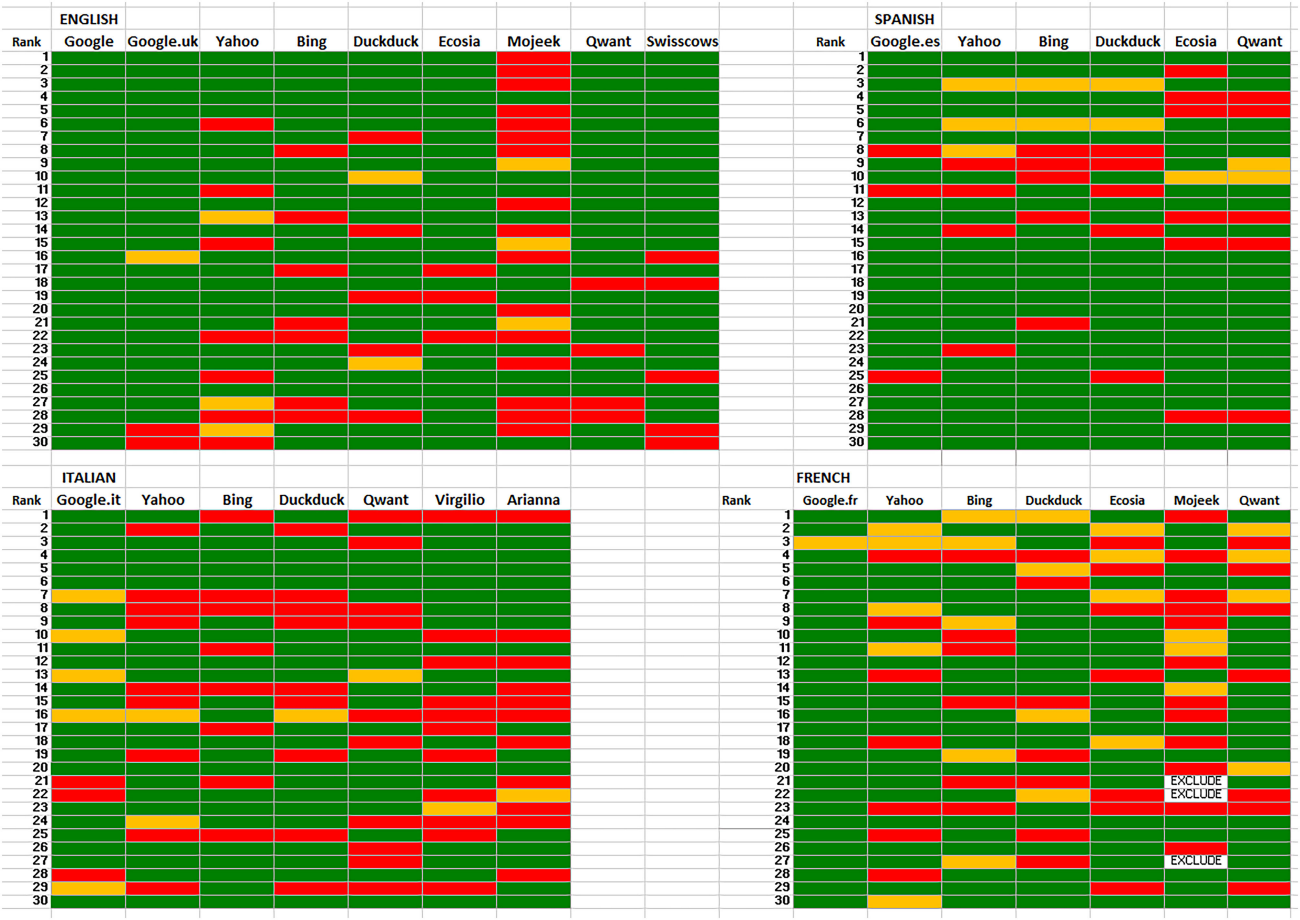
Stance on vaccines in webpages returned by different search engines in four languages. The top 30 webpages returned in the SERPs are shown. Green, vaccine-positive, yellow, vaccine-neutral, red, vaccine-negative.

Figure 1 shows the ranking of positive (green), neutral (yellow), and negative (red) websites returned by the different search engines in English, French, Italian, and Spanish.

Because the purpose of this study was to assess the ranking of misinformation, we compared the frequency of vaccine-negative webpages across the different search engines. Table 2 shows that Google is consistently returning less misinformation, although the Italian and Spanish versions of Google, as well as its UK English version, returned more vaccine-negative webpages than the English-US version (Google.com). Other search engines return more vaccine-negative webpages with some, like Mojeek in English and French or Arianna and Virgilio in Italian, more likely to rank higher webpages with misinformation.

**Table 2:** Vaccine-negative webpages in different SERPs.

	**English-UK**	**Italian**	**Spanish**	**French**
Google.com	0	–	–	–
Local google	2	3	3	0
Yahoo	7^*^	9	4	7^*^
Bing	7^*^	8	5	6^*^
Duckduckgo	5^*^	9	5	7^*^
Ecosia	3	–	6	7^*^
Qwant	4	10	5	7^*^
Mojeek	16^*^	–	–	12^*^
Swisscows	5^*^	–	–	–
Arianna	–	11	–	–
Virgilio	–	11	–	–

*Data represent the number of vaccine-negative pages (30 webpages for each SERP)*.

* *Significantly different from Google in the respective language (or Google.com for English-UK) by a two-tailed Fisher's test corrected for multiple comparison using the method of Benjamini, Krieger, and Yekutieli at a false discovery rate of 5%*.

In English, the frequency of vaccine-negative webpages in Yahoo, Bing, Duckduckgo, Swisscows, and Mojeek was significantly higher than in Google.com, with Mojeek also significantly higher than Google.co.uk. In Italian, all SERPs had a higher proportion of vaccine-negative webpages compared with Google.it, but this was not statistically significant, although it was if compared with international English google.com (5% FDR). The two Italian-only search engines (Virgilio and Arianna) returned the highest number of negative pages in Italian.

In French, all search engines returned a significantly higher number of vaccine-negative webpages than Google in French. In Spanish, there were, on average, less vaccine-negative results. All the search engines tested had a higher proportion of vaccine-negative results but this was not statistically significant when compared with local Google or with Google.com.

A common feature was that the SERPs of all search engines providers contained a higher proportion of vaccine-negative results than those obtained from Google.com. However, even the localized versions of Google (Google.co.uk, Google.it and Google.es) returned more negative pages than the US/international English Google.com.

## Discussion

The results indicate that currently privacy-enhancing search engines often give more visibility to webpages promoting vaccine hesitancy or with a clear anti-vaccine position than Google. This is in agreement with the findings from a recent study by the Economist which analyzed 175,000 news links returned by Google to demonstrate that the search engine's algorithm favors trustworthy publications (19). Reputation and trustworthiness are key factors included in Google's ranking algorithm. In 2019, Google published its search quality evaluation guidelines,^3^ which define webpages containing information that may affect the users' health or financial stability, as “your money your life” (YMYL) pages. These guidelines reveal that when rating YMYL webpages, Google looks at the three criteria of Expertise-Authority-Trustworthiness (E-A-T) and states:

“High E-A-T information pages on scientific topics should be produced by people or organizations with appropriate scientific expertise and represent well-established scientific consensus on issues where such consensus exists.”

Thus, in the case of Google, the bandwagon heuristic mechanism of assessing medical information credibility is working in favor of promoting IQ. This is perhaps because those responsible for driving up the “reputation” of specific websites by, for example, linking to them, have higher levels of eHealth literacy and therefore act as pseudo-gatekeepers protecting those with lower eHealth literacy from poor IQ results by ensuring that websites providing inaccurate online health information have a low ranking in the search results.

In the case of privacy-preserving engines, this appears not to be working—potentially because they do not track “reputation” factors, which could be seen as proxies for IQ and credibility, e.g., clicks and bounce-rate, and so have no gatekeepers (pseudo or otherwise) working to limit the circulation of misinformation. To our knowledge, the algorithms used by these “alternative” search engines are not public—Qwant states that they use their own algorithms^4^—but despite this, we found a large overlap between the SERP of Qwant and those of Bing and Ecosia. Likewise, Ecosia and Swisscows showed a similar overlap (up to 70%) with Bing, while Ecosia and Qwant often had a complete overlap. This means that it is not possible to check what factors determine the outcome of the decision making processes of the search engines algorithms and, hence, identify those elements that contribute to circulate misinformation.

It should be mentioned, however, that also non-privacy-savvy search engines, like Bing or Yahoo, often returned more vaccine-negative results than Google, also stressing that IQ is independent on privacy policy. More importantly, even localized versions of Google (UK English, Italian, and Spanish) returned more vaccine-negative results than Google.com or the one in French, indicating that many factors, including the use of non-English language and/or localization can affect the IQ of the results.

Even without being able to check the exact mechanisms for the overall poorer results, the results suggest that, currently, decisions made by search engines to prioritize privacy preservation may have a negative impact on the IQ of results returned to users in health contexts. The pledge to provide independent and unbiased results or not to promote or hide websites based on political or moral interests can be seen as ethically ambiguous, in view of the potential consequences of pointing to scientifically unsound health information.

Medical ethics requires that patients give informed consent before treatment and must, therefore, be informed accurately of the risks and benefits associated with treatment, something that is not possible if the search engine provider used by an individual is returning results with low IQ. From this consequentialist perspective, it is inherently unethical choosing not to interfere with a search engine's ranking algorithm to ensure “manually” that results of higher IQ are prioritized, while those of lower IQ are suppressed. This builds on arguments already made in the wider literature about algorithm ethics (20, 21). For example, the Association for Computing Machinery (ACM) code of ethics and professional conduct includes, as a general principle, “avoid harm” along with that of “respect privacy” (22), which aligns with the Hippocratic oath. Specifically, the implication that the current design of privacy-preserving search engine algorithms underestimates the need for evaluation of IQ fits into the wider discussion about algorithmic design and how to ensure design decisions are made to protect and incorporate key values such as IQ. It is necessary, but insufficient, to design search engine algorithms that index purely on “relevance,” they must also be designed to index on quality. The challenge lies in the ability to do this in a way that balances the need to accept different perspectives (particularly those that are rooted in different cultural, religious, or social ideals), while also filtering for IQ. Supporters of such arguments, including the authors, note that this requirement necessitates making the workings of search engine algorithms more transparent (23) to ensure their ethical compliance.

Providing information on vaccines that is based on misinformation or disinformation (including studies whose data or conclusions have been shown to be wrong) is a deceptive practice, which goes against the basic tenets of medical and business ethics (24). This is in line with those who argue that the promotion of “alternative medicine” is unethical because it lacks the evidence and transparency of clinical efficacy and should be considered “false advertising” (25). Online service providers have a moral responsibilities to ensure that users access health information that is scientifically validated (26). Misinformation and disinformation concerning healthcare circulating on the internet can have severe consequences and lead to widespread harm. Consider the example of South African president Mbeki, who delayed introducing anti-retroviral drugs in favor of alternative medicine based on information obtained from HIV-denialist internet websites, a decision estimated to have resulted in over 300,000 deaths (27). More recently, a cancer patient died in China after following an alternative, non-approved therapy they found using a search engine (28). In response, Chinese authorities issued new rules that require search engines to provide “objective, fair, and authoritative results.”

Another important aspect of IQ is its role in the context of informed consent, which is a central aspect of medical ethics, along with right to privacy and minimizing harm. It has been pointed out by Shahvisi (29) that to be informed about a treatment means not only to have knowledge but also to have an understanding of the treatment, and understandability is one of the basic dimensions of IQ (30). This raises the issue of the responsibility of search engines in the context of the existing health information to make informed and autonomous choices. If people make autonomous decisions based on the information obtained on the Internet (something that is often incorrectly called “doctor google” effect), one may argue whether search engines have a responsibility to provide high quality information. This is clearly a new challenge in medical ethics, and has been discussed, for instance, in the context of online information on medical tourism (31). The efforts made by the big internet companies, from search engines to social media, to pay particular attention to the IQ of health information indicates that they are well aware of the responsibility that comes with their role.

Focusing on data privacy without addressing the aspect of health IQ and its role in informed consent is a deficiency in the smaller search engines that would ultimately impact on the process of informed consent. This study, although limited to the information on vaccines, highlights an unregulated gray area for which search engines and regulators should be ethically responsible and that will need being addressed.

Moral responsibility follows on the level of harm that misinformation and disinformation on healthcare may cause. It could be argued that there is a greater onus on Google—and other commercial search engine providers—than on alternative search engines to take these considerations into account when designing or interfering with algorithms for the purposes of promoting ethical compliance, given their larger market share. Google currently has over 90% of the worldwide market share^5^ and therefore has the potential to indirectly “cause harm,” through the potential promotion of low IQ webpages on vaccinations, to a great many more people than any of the alternative providers.

However, studies have shown that those who hold anti-authoritarian views, openness to (potentially) controversial opinions, and an interest in alternative medicine are, in some cases, already more likely to hold vaccine-hesitant beliefs (17). These are also the individuals who are most likely to use alternative search providers given their sensitivity to privacy and tracking concerns. Therefore, while the alternative providers might reach a proportionally smaller audience—they are reaching an audience that is already more receptive to anti-vaccine information (32) and therefore more vulnerable to its effects. In other words, unless the alternative providers take steps to rank vaccine-related search results according to IQ, they may cause greater harm to those they do reach, meaning that the net negative impact is still greater even though the number of individuals they reach is smaller.

## Conclusion

Our analysis shows that while it may well be technically possible to design a search engine that manages to balance privacy-preservation with the promotion of high IQ material, this is currently not the case. The current relationship between privacy-preserving design features of search engines and the IQ of the results they return is inverse (although not proportionally). In instances where this can have harm public health, as in the example we have provided of the promotion of anti-vaccine misinformation, not intervening to alter the design of the algorithm—even if this means sacrificing some degree of user privacy—can lead to severe harm for a large population of users and is, therefore, unethical.

Designing a search engine that is privacy savvy and avoids issues with filter bubbles that can result from user-tracking may be a good thing, and in fact this aspect is, at least in part, regulated from the perspective of data protection—which is primarily interpreted as data security rather than data privacy. Our study suggests that this is necessary but insufficient, and instead mechanisms should be developed to test search engines from the perspective of IQ (particularly for YMYL webpages) before they can be deemed trustworthy providers of health information.

## Data Availability Statement

All datasets generated for this study are included in the article/Supplementary Material.

## Author Contributions

PG and MG designed the research. PG, PB, GC, AC, MG, MN, AP-B, PS, and TV performed the research and analyzed the data. PG, JM, MT, and LF analyzed the data and wrote the paper. All authors contributed to the article and approved the submitted version.

## Conflict of Interest

The authors declare that the research was conducted in the absence of any commercial or financial relationships that could be construed as a potential conflict of interest.

## Footnotes

^1^Misinformation is incorrect information that stems from human error e.g., a lack of fact checking, whereas disinformation is purposefully and deliberately incorrect. Both matter in this context because whilst the original 1998 study may have been an example of misinformation, it is possible that maleficent actors are now purposefully spreading disinformation for the purpose of undermining public health.

^2^eHealth literacy is defined by Norman and Skinner (7) as the ability to seek, find, understand, and appraise online health information and apply the knowledge gained to addressing or solving a health problem. This is why we use the term eHealth literacy even though this article focuses exclusively on the IQ of online health information —not broader sources of eHealth information such as electronic health records or wearable devices.

^3^https://static.googleusercontent.com/media/guidelines.raterhub.com/en//searchqualityevaluatorguidelines.pdf

^4^https://medium.com/qwant-blog/web-indexation-where-does-qwants-independence-stand-8eab4f7856f8 (Archived at: https://web.archive.org/web/20190627090553/https://medium.com/qwant-blog/web-indexation-where-does-qwants-independence-stand-8eab4f7856f8).

^5^https://gs.statcounter.com/search-engine-market-share
